# Efficacy of a Web-Based Computer-Tailored Smoking Prevention Intervention for Dutch Adolescents: Randomized Controlled Trial

**DOI:** 10.2196/jmir.2469

**Published:** 2014-03-21

**Authors:** Sanne de Josselin de Jong, Math Candel, Dewi Segaar, Henricus-Paul Cremers, Hein de Vries

**Affiliations:** ^1^School for Public Health and Primary Care (CAPHRI)Department of Health PromotionMaastricht UniversityMaastrichtNetherlands; ^2^Trimbos Institute (Netherlands Institute of Mental Health and Addiction)UtrechtNetherlands; ^3^STIVORO Dutch Expert Centre on Tobacco ControlDen HaagNetherlands; ^4^School for Public Health and Primary Care (CAPHRI)Department of Methodology and StatisticsMaastricht UniversityMaastrichtNetherlands

**Keywords:** computer tailoring, Web-based intervention, Internet, smoking prevention, smoking initiation, adolescents, randomized controlled trial

## Abstract

**Background:**

Preventing smoking initiation among adolescents is crucial to reducing tobacco-caused death and disease. This study focuses on the effectiveness of a Web-based computer-tailored smoking prevention intervention aimed at adolescents.

**Objective:**

The intent of the study was to describe the intervention characteristics and to show the effectiveness and results of a randomized controlled trial. We hypothesized that the intervention would prevent smoking initiation among Dutch secondary school students aged 10-20 years and would have the largest smoking prevention effect among the age cohort of 14-16 years, as smoking uptake in that period is highest.

**Methods:**

The intervention consisted of a questionnaire and fully automated computer-tailored feedback on intention to start smoking and motivational determinants. A total of 89 secondary schools were recruited via postal mail and randomized into either the computer-tailored intervention condition or the control condition. Participants had to complete a Web-based questionnaire at baseline and at 6-month follow-up. Data on smoking initiation were collected from 897 students from these schools. To identify intervention effects, multilevel logistic regression analyses were conducted using multiple imputation.

**Results:**

Smoking initiation among students aged 10-20 years was borderline significantly lower in the experimental condition as compared to the control condition 6 months after baseline (OR 0.25, 95% CI 0.05-1.21, *P*=.09). Additional analyses of the data for the 14-16 year age group showed a significant effect, with 11.5% (24/209) of the students in the control condition reporting initiation compared to 5.7% (10/176) in the experimental condition (OR 0.22, 95% CI 0.05-1.02, *P*=.05). No moderation effects were found regarding gender and educational level.

**Conclusions:**

The findings of this study suggest that computer-tailored smoking prevention programs are a promising way of preventing smoking initiation among adolescents for at least 6 months, in particular among the age cohort of 14-16 years. Further research is needed to focus on long-term effects.

**Trial Registration:**

International Standard Randomized Controlled Trial Number (ISRCTN): 77864351; http://www.controlled-trials.com/ISRCTN77864351 (Archived by WebCite at http://www.webcitation.org/6BSLKSTm5).

## Introduction

### Background

Of every three young smokers, one will die as a result of their tobacco use [1]. The overwhelming majority of smokers first begin to smoke during adolescence, the period in which youngsters are most vulnerable to social influences, tobacco product marketing, and risky behavior [2,3]. In fact, 88% of all first use occurred by age 18 and 99% of all adult smokers started smoking by the age of 26 [1,4].

In adolescents, nicotine dependence develops rapidly during experimentation, often before adolescents start smoking on a daily basis [5]. Early onset of tobacco use is associated with subsequent heavier smoking and contributes to greater rates of addiction [4,5]. Moreover, the younger children start smoking and persist in the habit as adults, the greater the risk of getting lung cancer and other smoking-related diseases [2,4]. To end the tobacco epidemic, it is therefore critical to prevent smoking onset among youngsters [1].

Several effective, mostly school-based, adolescent smoking prevention programs have been developed [6,7], including in the Netherlands [8-10], with positive program effects on smoking behavior sometimes lasting for up to 15 years [11]. However, doubts exist concerning sustained effects in the years following program delivery [6,12]. School-based programs encounter several challenges in implementation, including limited time and inadequate training for teachers [13-15]. By providing easily accessible and standardized information, computer-based interactive interventions have the potential to overcome these implementation challenges [16]. Moreover, computer-based interventions can also be used to reach youngsters in an out-of-school setting [17,18]. They appear an attractive method to engage young people in smoking prevention and cessation [19,20]. Focus group results, conducted among 15-17 year old Dutch students, demonstrated that the Internet is the most desired medium for education about smoking. Adolescents preferred clear, interactive, and personal information regarding this topic [21]. Web-based computer-tailored interventions fit this need.

Computer-tailored interventions provide feedback adapted to the user’s individual characteristics and needs [22]. By increasing personal relevance, tailored messages are more likely to be read, thoughtfully considered, and influence beliefs and behaviors [22-26]. Computer-tailored interventions can reach large groups of people in a cost-effective way [25,27,28], and users can take part in the intervention in private at any preferred time [29]. An additional advantage of computer-tailored interventions for schools is that, due to a semiprivate computer environment, students can be more willing to disclose personal information and smoking status [30]. Web-based computer-tailoring has proven to be successful in influencing health behaviors like nutrition and physical activity, among both adults [27,29,31] and adolescents [32,33]. Additionally, multiple studies have demonstrated the effectiveness of Web-based computer-tailored interventions for the promotion of adult smoking cessation [24,34,35].

Few studies have focused on computer-based tailored programs addressing adolescent smoking prevention. Both Prokhorov and colleagues [16,30] and Buller and colleagues [18] evaluated computer-based tailored smoking prevention programs for adolescents, consisting of multiple sessions delivered over 6 weeks. Although the results suggest that these tailored programs may be beneficial for the prevention of smoking, the researchers reported problems with recruitment and retention of adolescents. They stress the need for shorter interventions [18] that are theory-based, technologically advanced, and tailored to the needs of adolescents [30].

This paper focuses on a Web-based smoking prevention and cessation program aimed at Dutch adolescents, called “Smoke Alert”, which consisted of a Web-based questionnaire and fully automated, computer-tailored feedback. Smokers were provided feedback messages about how to stop smoking and non-smokers could learn how to refrain from smoking. The Smoke Alert program addressed both smoking cessation and prevention, as adolescents in schools for this age category can be both smokers and non-smokers. The intervention presented in this study was an improved version of the Smoke Alert program that was described in an earlier study and had shown positive effects on smoking cessation [36]. This paper addresses the effectiveness of the intervention for the prevention of smoking.

### Objectives

The main aim of this paper is to describe the intervention characteristics and to show the results of the randomized controlled trial on its effectiveness for the prevention of smoking among Dutch adolescents. This trial was conducted among students ranging from 10-20 years of age in order to detect whether implementation could be recommended for different age groups, since usage statistics showed that a wide age range of students participated in the previous version of the Smoke Alert program. We hypothesized that smoking initiation rates would be lower in the experimental condition at 6-month follow-up, as compared to the control condition (hypothesis 1). By targeting social influences and providing skills for refusing cigarettes, we expected the smoking prevention program to be most effective for adolescents in a context in which some of their peers already smoke [18]. Smoking initiation in the Netherlands is highest between the ages of 14 and 16, with uptake levels ranging from 7% at age 14 to 23% at age 16 respectively [37]. Consequently, we expected the program to have significant effect in particular in this specific at-risk age group (hypothesis 2). Finally, we explored whether gender and baseline education level of adolescents were potential moderators in the present study.

## Methods

### Design

Intervention effectiveness was studied by means of a cluster randomized controlled trial and encompassed the implementation of Smoke Alert in the experimental condition (at school). The intervention was being tested against a no-intervention control condition. Allocation ratio was 1:1 and respondents from both conditions filled out a Web-based questionnaire at baseline and at 6-month follow-up, assessing smoking behavior, intention to start smoking, age, gender, and educational level. The trial is registered in the ISRCTN Register (ISRCTN77864351).

### Participants and Procedure

Participants in the present study were students from secondary schools in the Netherlands. The eligibility criteria for participants were: age between 10 and 20 years; having computer/Internet literacy; having sufficient command of Dutch; no previous exposure to the earlier version of Smoke Alert [36]; and being a non-smoker or former smoker. During the spring of 2011, 1380 secondary schools throughout the country were approached by sending a letter to their principals. The principals were asked to hand out the attached flyers to their teachers, inviting them to make use of a free computer-tailored smoking intervention in their classrooms, as part of an effectiveness trial. Local health departments assisted in recruiting schools through announcements on websites and in newsletters. Teachers were invited to digitally sign up for participation to the Smoke Alert intervention. After subscription, teachers received a letter with more extensive information about the purpose, design, and planning of the effectiveness study. Furthermore, a letter to inform the students’ parents was attached. Teachers were requested to schedule 30 minutes, between 9 May and 10 June 2011, for the students to complete the Web-based questionnaire in the classroom. In contrast to the baseline assessment, which took place at school, the students were invited by email for the 6-month follow-up measurement. Students who did not supply a valid email address at baseline were excluded from participation in the follow-up measurement. To stimulate response, students were told that they could win an iPod or cinema voucher by participating in the follow-up assessment. Respondents in the control condition were given the opportunity to obtain computer-tailored advice after they filled out the follow-up questionnaire.

### Intervention

**Figure 1:**
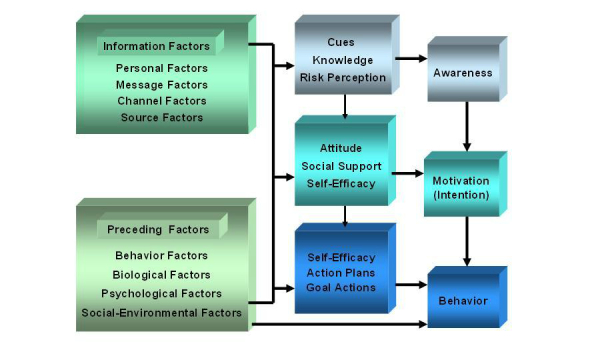
The I-Change Model [38,39].

The Smoke Alert program was based on the I-Change Model, or the Integrated Model for exploring motivational and behavioral change [38,39]. According to the I-Change Model, behavior (eg, smoking behavior) is influenced by awareness factors (knowledge, risk perceptions, and cues to action), motivational factors (attitudes, social influence beliefs, and self-efficacy), and action factors (action plans and goal actions) (Figure 1).

The previous version of Smoke Alert [36] was revised on the basis of focus group discussions with adolescents suggesting improvements such as the use of avatars, different Web design, and less extensive feedback messages. In order to increase recruitment and retention of adolescents, the revised version contained a combination of textual information with other (content-related) elements like graphics and animated videos [40-42].

The questionnaire and content of the feedback messages of Smoke Alert were updated versions of previously used questionnaires and feedback, derived from evidence-based interventions on smoking prevention and cessation [9,10,17,43]. Pilot tests revealed that the questionnaire should not be too long, resulting in a questionnaire that focused on assessing sociodemographics (age, gender, and education level), intention to start smoking, and motivational determinants.

To measure intention to start smoking, students were asked to select a statement that best described their situation, with options ranging from “I know for sure I won’t ever start smoking” to “I think I will start smoking within 1 month”. Three social cognitive concepts were measured according to the I-Change Model: namely, attitude towards smoking, perceived social influence, and self-efficacy not to smoke. Attitudes were assessed by 9 items that measured the pros and cons of smoking, for instance: “If I smoked, I would feel more confident”, “If I smoked, it would cost me a lot of money”, etc. Perceived influences from the social environment were measured by 2 items that assessed social modeling. Self-efficacy was measured with 6 items via which students could indicate how sure they were that they could remain a non-smoker in certain situations. These situations can be divided into 2 types: stressful situations (eg, feeling nervous) and social situations (eg, at a party, when friends smoke) [38]. Finally, non-smokers were asked to indicate to what extent they planned on using certain strategies when someone would offer a cigarette, for instance, using a clear “no” statement, stating the reason for refusing the cigarette, walking away, etc.

The respondents used their unique log-in information, provided by their teachers, to access the intervention website at school. Students in the experimental condition received their feedback on the computer screen immediately after filling out the questionnaire. The advice consisted of a home page, containing an introduction and a 30-second animated video, as well as several subpages, each providing feedback on a specific determinant (for a screenshot of the home page, see Multimedia Appendix 1). Facts and figures were depicted on the right and left sides of the pages, also tailored to the answers of the students. The introduction consisted of a personal greeting that contained the name of the student, followed by a confirmation of their smoke-free status and intention to start smoking. Students were praised for being a non-smoker. The animated video presented a male or female avatar and focused on reasons to refuse a cigarette (eg, “Why would I take the cigarette? My girlfriend/boyfriend would never want to kiss me anymore”). The video content was based on principles of social cognitive theory [44], also used in a previous booklet-based version in the Netherlands and Romania [8,43], and focused on social influences. The subpages of the advice addressed the psychosocial determinants.

The first subpage was dedicated to beliefs about smoking (ie, attitude). The students’ beliefs were considered as a balance indicating whether he or she perceived more or less advantages than disadvantages of smoking. The students’ opinion of each belief was stated and commented on. These messages had the general intention of countering beliefs about the positive effects of smoking (eg, smoking will make me feel relaxed, smoking will make me popular) and to strengthen beliefs about the negative effects (eg, smoking will cost me a lot of money). The second subpage addressed the perceived social influence. Based on students’ answers, they were informed about the negative influence of smokers in their environment. When the student indicated having a lot of smokers in their environment, the idea of smoking as a “normal activity” was counteracted by stating that the majority of people in the Netherlands do not smoke. When most people in the environment of the student were non-smokers, the feedback confirmed that smoking is not the norm in the Netherlands. The third subpage was dedicated to self-efficacy. For situations where the student expected difficulties in remaining a non-smoker, strategies were offered to help the student to get through these situations without initiating smoking (eg, thinking about the reasons for being a non-smoker). The final subpage focused on action plans. The feedback reflected on every action plan the student had indicated he or she would use in situations where someone would offer a cigarette. Examples of action plans were provided when a student was not planning to use a certain action plan. The main message regarding action plans was: by preparing yourself for the situation when someone offers you a cigarette, you will be more confident and it will be easier to refuse the cigarette.

Examples of the computer-tailored feedback messages are provided in Table 1. A copy of the advice (a PDF file) was sent when an email address was voluntarily provided. This way, the students could re-read or print their advice at home.

**Table 1:** Examples of feedback messages [36].

Feedback type	Message
Intention feedback	*You don’t smoke. That’s great! But...do you really want to try a cigarette in the future? That would be a pity. In this advice, you’ll discover what you really think about smoking.*
Attitude feedback	*Your answers show that you see a lot of advantages of smoking. That can make it difficult to remain a non-smoker. Let’s have a look at your answers.*
Social influence feedback	*Almost none of your friends smoke. However, you mentioned about half of the people around you smoke. Maybe you feel like smoking is normal. But that’s not true. Most people in the Netherlands do not smoke. Did you know that almost three-quarters of Dutch citizens do not smoke? So only 1 out of 4 people is a smoker.*
Self-efficacy feedback	*You find it difficult not to smoke when you’re at a party. Why would you start smoking? Think about your reasons for being a non-smoker. Smoking with others at a party doesn’t make you better company for your friends. That has nothing to do with cigarettes. Your friends like you for who you are. They probably think it’s good that you don’t smoke.*
Action plans feedback	*There’s one thing you’ll do when someone offers you a cigarette. You’ll just say no and explain why you don’t want it. Very good. For some youngsters, it’s hard to say no. It’s also good that you’ll explain why you don’t want to smoke. This way, they won’t offer you a cigarette again. Think in advance what you would do when they keep pushing you. That can make you feel more confident.*

### Measures

#### Outcome Measurements

The primary outcome measure was smoking behavior defined as smoking at least occasionally. Respondents were asked to pick a statement that best described them out of 9 smoking-related statements [9,17,36,45]. They were categorized as non-smokers if they selected one of the following statements: (1) “I have never smoked a puff”, (2) “I have tried smoking but I do not do this anymore”, (3) “I have stopped smoking. I used to smoke less than once a week”, or (4) “I have stopped smoking. I used to smoke more than once a week”. Respondents were categorized as smokers if they selected one of the following statements: (5) “I try smoking sometimes”, (6) “I smoke less than once a month”, (7) “I smoke at least once a month, but not weekly”, (8) “I smoke at least once a week, but not daily”, or (9) “I smoke daily”. To quantify the intervention effects on smoking initiation, we assessed the percentage of baseline non-smokers that indicated to smoke at follow-up.

#### Baseline Measurements

Intention to start smoking was measured by asking students to select a statement that best described their situation, with the following response options: (1) “I know for sure I won’t ever start smoking”, (2) “I think I won’t ever start smoking”, (3) “I think I will start smoking in the future”, (4) “I think I will start smoking within 5 years”, (5) “I think I will start smoking within 1 year”, (6) “I think I will start smoking within 6 months”, and (7) “I think I will start smoking within 1 month”. Adolescents were also asked to report their age (in years), gender (1=“male”, 0=“female”) and educational level: high (senior general secondary education / pre-university education=1) or low (practical education / lower secondary professional education=0).

### Sample Size

Power analysis was based on the assumption that 2% of the experimental condition would initiate smoking 6 months after baseline, whereas among the control condition the national prevalence rate of ever smoking was expected to increase by 7% at the age of 15, the expected mean age at follow-up. To be able to detect this difference with a power of .80 at 5% significance level (two-sided testing), assuming that 95% of the schools in the control condition have uptake rates between 0.8% and 56%, corresponding with an intraclass correlation (ICC) of .34 on the logit scale, 54 schools and 702 non-smokers should be included in the study. Accounting for the efficiency loss due to unequal amounts of students per school, the number of schools was raised by 10% [46], resulting in 60 schools and 780 non-smokers. After adjusting for a potential 50% dropout at student level at 6 months [36], at least 1560 non-smokers had to be included in this study. In 2011, the national prevalence rate of ever smoking among Dutch adolescents aged 10-19 years was 37% [37]. Of these ever smokers, 47% had already stopped smoking, resulting in an expected smoking prevalence rate of 20%. To include at least 1560 non-smokers in the study, a total of 1950 students was required.

### Randomization

The schools were randomly assigned to the experimental or control condition. Randomization was performed automatically by computer software that was developed specifically for the execution of Web-based computer-tailored programs [47]. The teachers were informed by email about their allocation to either the experimental or control condition, with unique log-in information for each student attached. The teachers who were allocated to the control condition were told that their students could take part in the intervention 6 months later, after filling out the baseline and follow-up questionnaire. This way, all teachers who signed up could be offered the Smoke Alert program.

### Statistical Methods

All analyses were done using MLwiN (multilevel modelling for Windows), since adolescents were nested within schools. Ignoring this nesting structure may inflate type I errors and lead to too narrow confidence intervals for treatment effects [48]. Previous Dutch studies on smoking prevention at primary schools [17] and smoking cessation at secondary schools [36] also used this type of analysis. To check whether the randomization was successful, both conditions were compared on age, gender, educational level, and intention to start smoking. Dropout was checked using multilevel logistic regression analysis with attrition at post-test as outcome, and baseline demographic variables and intention to start smoking as predictors. Interaction terms of predictors with treatment condition were included in the model to analyze whether predictors for dropout differed by condition. Differences between the conditions on smoking initiation were analyzed by multilevel logistic regression analysis. Demographic variables and significant baseline differences were entered as covariates. Interactions of these covariates with treatment condition were also included to examine inequalities in the effects of the intervention on smoking initiation. Interactions with a *P* value higher than .05 were deleted stepwise. Effects of covariates and the intervention were considered significant if *P*≤.05. To accommodate missing values in the effect analyses, the multiple imputation procedure in MLwiN was employed, the results being based on 50 imputed datasets. This procedure saves cases for the analysis and can be considered an intention-to-treat analysis. Analysis under multiple imputation is valid when the data are missing at random [49], that is, when the missingness only depends on variables included in the analysis. In this case, it is considered to be the best method available for imputing missing values [49].

### Ethics

Students’ participation in both conditions was voluntary, respondents were guaranteed anonymity, and it was explained that they could withdraw participation at any time. This study was part of a larger study on the effectiveness of the Smoke Alert study for which ethical clearance was obtained [36].

## Results

### Study Recruitment and Sample Characteristics

In total, 89 schools signed up for participation, resulting in a total of 10,500 students. At baseline, 83 out of 89 schools responded to the questionnaire, 4 schools indicated that they no longer had time to participate, and 2 schools did not explain their non-response to the baseline questionnaire. A total of 6078 students completed the baseline questionnaire, 1099 did not meet the inclusion criteria, resulting in a total of 4979 non-smokers that remained for participation in the follow-up measurement.

Mean age of the respondents at baseline was approximately 14 years (SD 1.1), with age ranging between 10 and 20 years (Table 2). Of the 4979 participants, 2518 (50.57%) were male and 2744 (55.11%) were students at a lower level of education. At baseline, no significant differences between the experimental and control condition were observed (*P*>.05).

**Figure 2:**
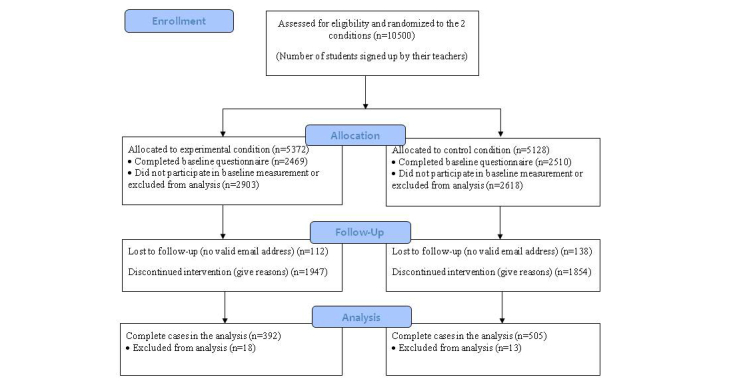
Participant flow chart.

The CONSORT flowchart (Figure 2) shows the flow of respondents from enrollment in the study to allocation to the experimental (E) and control (C) condition, and whether they were included in the analysis. Of the 4979 participants that completed the baseline questionnaire (E: n=2469; C: n=2510), 4729 respondents supplied a valid email address and were invited by email to participate in the follow-up survey. After 2 email reminders, 712 participants completed the follow-up questionnaires. Non-responding students received an invitation by email to briefly indicate their current smoking status by selecting a statement that best described their behavior. This strategy resulted in a final sample size of 897 adolescents with complete data (E: n=392; C: n=505) from 64 schools at 6-month follow-up (ie, response rate 18%).

Attrition analysis showed that lower educated students were significantly more likely to drop out compared to higher educated students (OR 0.37, 95% CI 0.19-0.70, *P*=.002), and male students were more likely to drop out than female students (OR 1.77, 95% CI 1.11-2.82, *P*=.02). Furthermore, dropout was higher among respondents with a higher intention to start smoking (OR 1.37, 95% CI 1.03-1.82, *P*=.03). There were no significant differences (*P*>.05) regarding dropout between the experimental and control condition nor any significant interaction effects (*P*>.05) between covariates and the intervention factor.

**Table 2:** Baseline sample characteristics of non-smoking adolescents (n=4979), recruited in 2011.

Characteristic		Total (n=4979)	Experimental condition (E) (n=2469)	Control condition (C) (n=2510)
Age in years, mean (SD)		13.7 (1.1)	13.7 (1.0)	13.7 (1.1)
Male, n (%)		2518 (50.57%)	1220 (49.41%)	1298 (51.71%)
**Educational level, n (%)**				
	Low	2744 (55.11%)	1374 (55.65%)	1370 (54.58%)
	High	2235 (44.89%)	1095 (44.35%)	1140 (45.42%)
Intention to start smoking, mean (SD)		1.55 (0.7)	1.58 (0.7)	1.52 (0.7)

### Effects on Smoking Initiation at 6-Month Follow-Up in the Overall Population

Of the 392 students with complete data in the experimental condition, 15 (3.8%) initiated smoking 6 months after baseline. Of the 505 complete cases in the control condition, 28 (5.5%) initiated smoking. Table 3 shows the results of the regression model predicting smoking initiation at 6-month follow-up, employing multiple imputation. After adjusting for demographic variables and intention to start smoking, the experimental condition predicted smoking initiation close to significance (OR 0.25, 95% CI 0.05-1.21, *P*=.09) with students in the control condition reporting higher initiation, yielding borderline significant support for the first hypothesis. Another significant predictor of smoking initiation was intention to start smoking, with students with a higher intention being at higher risk. The ICC, reflecting the proportion of unexplained outcome variance that was accounted for by the schools, was .73 (*P*<.001), as obtained with the regression model in Table 3.

Interactions were added to this model to analyze whether the effect of the program was gender, education, and age dependent. No significant interactions were found regarding these covariates (*P*>.05).

**Table 3:** Predictors of smoking initiation at 6-month follow-up.

Predictor	OR	95% CI	*P* value
Intervention (1=yes, no=0)	0.25	0.05-1.21	.09
Gender (male=1, female=0)	1.21	0.63-2.32	.56
High educational level (1=yes, no=0)	0.53	0.20-1.37	.19
Age	0.16	0.94-1.42	.17
Intention to start smoking (never=1, within 1 month=7)	2.51	1.62-3.89	<.001

### Effects on Smoking Initiation at 6-Month Follow-Up Among 14-16 Year Olds

Next, in order to test our second hypothesis, an analysis was done for the age cohort of 14-16 years. There were 385 complete cases (E: n=176; C: n=209), with 24 students (11.5%) in the control condition reporting initiation compared to 10 students (5.7%) in the experimental condition. Table 4 shows the results of the analyses based on multiple imputation. After adjusting for demographic variables and intention to start smoking, condition predicted smoking initiation significantly (OR 0.22, 95% CI 0.05-1.02, *P*=.05). Similar to the analysis for the overall population, intention to start smoking was a significant predictor of smoking initiation (students with a higher intention being at higher risk). The ICC was .43 (*P=*.07).

**Table 4:** Predictors of smoking initiation at 6-month follow-up for students aged 14-16 years.

Predictor	OR	95% CI	*P* value
Intervention (1=yes, no=0)	0.22	0.05-1.02	.05
Gender (male=1, female=0)	1.69	0.75-3.84	.21
High educational level (1=yes, no=0)	0.43	0.11-1.63	.21
Age	2.09	1.11-3.94	.02
Intention to start smoking (never=1, within 1 month=7)	2.96	1.83-4.78	<.001

## Discussion

### Principal Findings

This paper describes a cluster randomized controlled trial examining the effectiveness of a computer-tailored intervention on smoking prevention, called Smoke Alert, aimed at adolescents. This trial was conducted among students aged 10-20 years in order to detect whether implementation could be recommended for a wide age range of students. We hypothesized that smoking initiation rates would be lower in the experimental condition at 6-month follow-up, as compared to the control condition. The results offered some support for our first hypothesis revealing that students in the control condition reported higher smoking initiation at 6-month follow-up. The results provided significant support for our second hypothesis, as the data for the 14-16 year age group showed a significant effect with lower smoking initiation rates in the experimental condition.

The results of this study support earlier findings that Web-based computer-tailored programs can be an effective tool in the prevention of smoking among youth [16-18]. Moreover, the results for the 14-16 year age group confirm the hypothesis of Buller and colleagues [18] that tailored smoking prevention programs targeting social influences and providing skills for refusing cigarettes will be most effective for adolescents in a context in which some of their peers already smoke. A similar conclusion was drawn from a recent Dutch smoking prevention program for 9-11 year-olds that did not reveal any effects, most likely because smoking has become less accepted leading to later onset rates among Dutch youth [50]. Based on our results, implementation of the Smoke Alert program is recommended, in particular, for students aged 14-16 years, when smoking uptake in the Netherlands is highest [37].

Smoking initiation was lower in the experimental condition among both higher and lower educated students. This is encouraging, since educational level is one of the strongest predictors of smoking behavior [51,52]. Also, in the Netherlands, smoking is more prevalent among secondary school students with a lower educational level [37]. Effective smoking prevention programs for lower educated students could reduce the gap in smoking prevalence between lower and higher educated students.

Schools serve as an important access point to reach many adolescents. Hence, it is recommended to incorporate the intervention within the regular curriculum at school. This way, even the least motivated adolescents will participate and complete the intervention. As has been noted previously, implementation challenges at school contribute to the decay of prevention program effects over time [13]. By providing easily accessible, standardized information in a semiprivate computer environment, we expect the Smoke Alert program to overcome these implementation challenges. Further research is needed to focus on effects of the Smoke Alert program in the years following program delivery.

### Limitations

There are several important limitations to consider in interpreting the results of this study. First, all measurements were self-reports. Biochemical validation may not be necessary or advisable in studies like the current study using Internet data collection without face-to-face contact [53]. In their review of tobacco cessation interventions for young people, Grimshaw and Stanton [54] noted that biochemical validation can affect recruitment and retention, and may not be a very sensitive measure of change in smoking behavior for irregular smokers. For these reasons, logistical constraints, and the fact that we promised anonymity, we did not perform biochemical verification. When confidentiality and anonymity are assured, adolescent self-reported smoking will lead to similar results as obtained by biochemical validation [55,56]. Hence, in the present study, the respondents were guaranteed confidentiality and were informed of the exclusion of their names and email addresses from the remaining answers [57].

Second, of the 1380 schools approached, only 89 agreed to participate, which may reveal an overall negative climate toward smoking prevention in the Netherlands and/or to participating in experimental studies. Most often, however, reasons for non-participation mentioned were lack of time and lack of interest, which is often the case in many schools in the Netherlands, since health promotion is not integrated into the Dutch school curriculum [10,58,59]. This clearly implies a need for health promoting policies to outline the need for adoption of evidence-based smoking prevention programs.

Third, we experienced high but equal loss to follow-up in both the experimental and control condition. The attrition at student level was 82% and outnumbered the expected dropout rate of 50%. High attrition is a well-known feature of many studies of eHealth interventions [60,61] and may be a threat to internal validity. In the current study, attrition risk may have been elevated because participants had to complete the follow-up questionnaire in their spare time, as compared to it being an activity at school [62]. Attrition analyses showed that dropout was selective. Dropout was higher among lower educated and older students and adolescents with a higher intention to start smoking. Since intention is an immediate antecedent of behavior [63], caution is warranted in generalizing the findings. Also, even though the effect analysis corrected for several covariates, the number of covariates was rather limited, implying that unobserved confounding may have occurred, thus biasing the results on the treatment’s effectiveness.

Handling dropout was performed using multiple imputation, thus preserving as many cases for analysis on the intervention effects as possible. Multiple imputation is considered the best method for imputing missing values [49], provided the missing values are missing at random. The current study did not allow time for qualitative assessment of missingness due to dropout, as we promised anonymity. The most likely explanation for non-participation, as voiced by their teachers, is that the students had a lack of interest in participating in a study, rather than reasons relating to their smoking behavior. It is important to gain insight into predictors of dropout and examine strategies to enhance engagement with Web-based interventions over time and reduce the excessive rates of attrition. Web 2.0 features, like allowing adolescents to manage, display, and share their data with peers, could be incorporated in order to attract, retain, and engage adolescents [62,64], although these hypotheses require additional research.

### Conclusions

Web-based computer-tailored interventions are a promising way of preventing smoking initiation among adolescents for at least 6 months, in particular among the age cohort of 14-16 years. The findings of the present study illustrate the need for smoking prevention programs beyond the 12-14 year age group that is traditionally targeted by these programs. Long-term assessment is needed to determine if the preventive effect of Web-based computer-tailored interventions is sustained in the years following program delivery.

## Multimedia Appendix 1

Home page showing computer-tailored feedback with animated video.

## Multimedia Appendix 2

CONSORT-EHEALTH checklist V1.6.2 [65].
