# Supplementary figures and images for: Efficacy of a Web-Based Computer-Tailored Smoking Prevention Intervention for Dutch Adolescents: Randomized Controlled Trial
Source: J Med Internet Res. 2014 Mar 21;16(3):e82. doi: 10.2196/jmir.2469 (PMC3978560; doi:10.2196/jmir.2469)

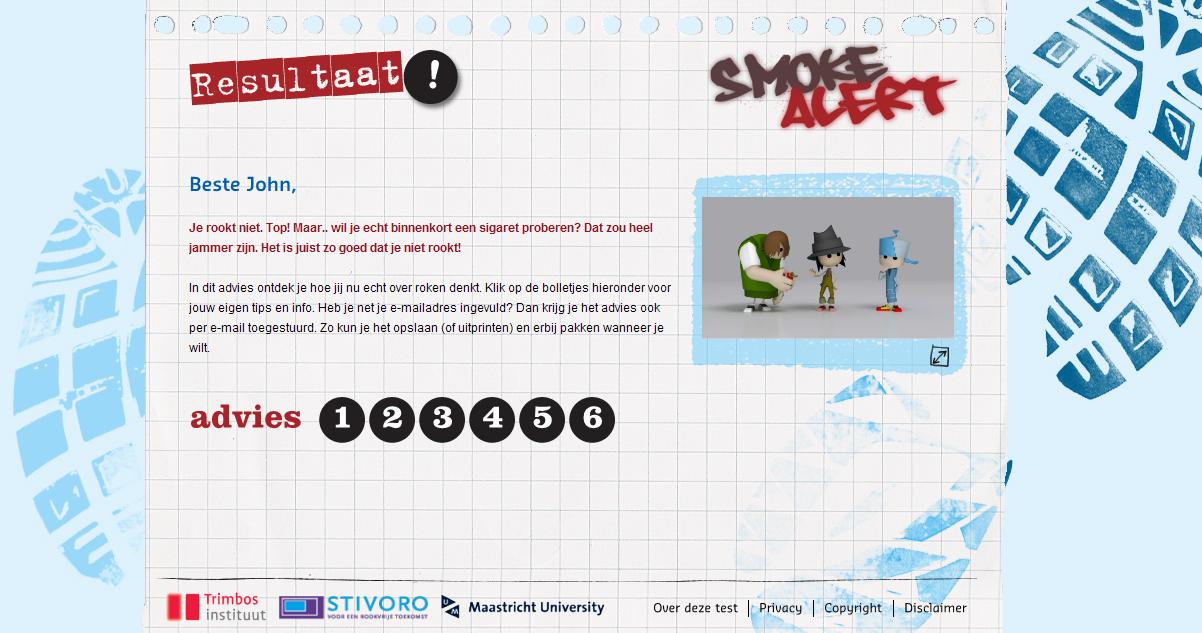

Supplement: Supplementary file 1 [file jmir_v16i3e82_app1.JPG]
