# Supplementary material for: Efficacy of a Web-Based Computer-Tailored Smoking Prevention Intervention for Dutch Adolescents: Randomized Controlled Trial
Source: J Med Internet Res. 2014 Mar 21;16(3):e82. doi: 10.2196/jmir.2469 (PMC3978560; doi:10.2196/jmir.2469)
Supplement: Supplementary file 2 [file jmir_v16i3e82_app2.pdf]

CONSORT-EHEALTH Checklist V1.6.2 Report

(based on CONSORT-EHEALTH V1.6), available at [http://tinyurl.com/consort-ehealth-v1-6].

2469

Date completed

1/29/2014 10:46:34

by

Sanne de Josselin de Jong

Efficacy of a web-based computer-tailored smoking prevention intervention for Dutch adolescents: A randomized controlled trial

TITLE

1a-i) Identify the mode of delivery in the title

"[...] web-based computer-tailored smoking prevention intervention [...]"

1a-ii) Non-web-based components or important co-interventions in title

n/a

1a-iii) Primary condition or target group in the title

"[...] for Dutch adolescents [...]"

ABSTRACT

1b-i) Key features/functionalities/components of the intervention and comparator in the METHODS section of the ABSTRACT

" The intervention consisted of a questionnaire and computer-tailored feedback on intention to start smoking and motivational determinants."

1b-ii) Level of human involvement in the METHODS section of the ABSTRACT

"[...] fully automated computer-tailored feedback [...]"

1b-iii) Open vs. closed, web-based (self-assessment) vs. face-to-face assessments in the METHODS section of the ABSTRACT

"[...] A total of 89 secondary schools were recruited via postal mail [...]"

1b-iv) RESULTS section in abstract must contain use data

"Smoking initiation among students aged 10-20 years was borderline significantly lower in the experimental condition as compared to the control condition 6 months after baseline (OR = 0.25, 95% CI = 0.05–1.21, P = .09). Additional analyses of the data for the 14-16 age group showed a significant effect, with 12% of the students in the control condition reporting initiation compared to 6% in the experimental condition (OR = 0.22, 95% CI = 0.05–1.02, P = .05). No moderation effects were found regarding gender and educational level." The number of participants is already mentioned in the method section (n=897).

1b-v) CONCLUSIONS/DISCUSSION in abstract for negative trials

n/a

INTRODUCTION

2a-i) Problem and the type of system/solution

"[...] To end the tobacco epidemic, it is therefore critical to prevent smoking onset among youngsters. [...] School-based programs encounter several challenges to implementation, including limited time and inadequate training for teachers [13-15]. By providing easily accessible and standardized information, computer-based interactive interventions have the potential to overcome these implementation challenges [16]. [...] Focus groups results, conducted among 15-17 year-old Dutch students, demonstrated that internet is the most desired medium for education about smoking. Adolescents preferred clear, interactive and personal information regarding this topic [21]. Web-based computer-tailored interventions fit this need. […] By increasing personal relevance, tailored messages are more likely to be read, thoughtfully considered, and influence beliefs and behaviors [22-26]. Computer-tailored interventions can reach large groups of people in a cost-effective way [25, 27, 28] and users can take part in the intervention in privacy at any preferred time [29]. An additional advantage of computer-tailored interventions for schools is that, due to a semiprivate computer environment, students can be more willing to disclose personal information and smoking status [30]."

2a-ii) Scientific background, rationale: What is known about the (type of) system

"[...] Web-based computer-tailoring has proven to be successful in influencing health behaviors like nutrition and physical activity, among both adults [27, 29, 31] and adolescents [32, 33]. Additionally, multiple studies have demonstrated the effectiveness of web-based computer-tailored interventions for the promotion of adult smoking cessation [24, 34, 35]. Few studies have focused on computer-based tailored programs addressing adolescent smoking prevention. Both Prokhorov and colleagues [16, 30] and Buller and colleagues [18] evaluated computer-based tailored smoking prevention programs for adolescents. [...] They stress the need for shorter interventions [18] that are theory-based, technologically advanced and tailored to the needs of adolescents [30]. This paper focuses on a web-based smoking prevention and cessation program aimed at Dutch adolescents, called Smoke Alert, that consisted of a questionnaire and computer-tailored feedback."

METHODS

3a) CONSORT: Description of trial design (such as parallel, factorial) including allocation ratio

"The main aim of this paper is to describe the intervention characteristics and to show the results of the randomized controlled trial on its effectiveness for the prevention of smoking among Dutch adolescents. This trial was conducted among students ranging from 10-20 years of age in order to detect whether implementation could be recommended for different age groups, since usage statistics showed that a wide age range of students participated in the previous version of the Smoke Alert program. We hypothesized that smoking initiation rates would be lower in the experimental condition at 6-month follow-up, as compared to the control condition (hypothesis 1). By targeting social influences and providing skills for refusing cigarettes, we expected that the smoking prevention program to be most effective for adolescents in a context in which some of their peers already smoke [18]. Smoking uptake in the Netherlands is highest between age 14 and 16, with uptake levels ranging from 7% at age 14 to 23% at age 16 respectively [37]. Consequently, we expected the program to have significant effect in particular in this specific at risk age group (hypothesis 2). Finally, we explored whether gender and baseline education level of the adolescents were potential moderators in the present study."

3b) CONSORT: Important changes to methods after trial commencement (such as eligibility criteria), with reasons

After two reminders, we sent out an email to the participants inviting them to briefly indicate their current smoking status by selecting a statement that best described their behavior. This way, we wanted to increase the response to the primary outcome measure (smoking behavior).

3b-i) Bug fixes, Downtimes, Content Changes

n/a

4a) CONSORT: Eligibility criteria for participants

"Participants in this study were students from secondary schools in the Netherlands. The eligibility criteria for participants were: age between 10 and 21; having computer/Internet literacy; having sufficient command of Dutch; no previous exposure to the earlier version of Smoke Alert [36]; and being a non-smoker or former smoker."

4a-i) Computer / Internet literacy

[...] having computer / Internet literacy [...]

4a-ii) Open vs. closed, web-based vs. face-to-face assessments:

"[…] 1380 secondary schools throughout the whole country were approached by sending a letter to their principals. [...] Local health departments assisted in recruiting schools through announcements on websites and in newsletters. Teachers were invited to digitally sign up for participation to the Smoke Alert intervention. After subscription, teachers received a letter with more extensive information about the purpose, design and planning of the effectiveness study. [...] In contrast to the baseline assessment, which took place at school, the students were invited by email for the 6-month follow-up measurement."

4a-iii) Information giving during recruitment

"The principals were asked to hand out the attached flyers to their teachers, inviting them to make use of a computer-tailored smoking intervention in their classrooms for free - as part of an effectiveness trial. [...] After subscription, teachers received a letter with more extensive information about the purpose, design and planning of the effectiveness study. Furthermore, a letter to inform the students' parents was attached."

4b) CONSORT: Settings and locations where the data were collected

"Teachers were requested to schedule half an hour, between 9 May and 10 June 2011, for the students to complete the web-based questionnaire in the classroom. In contrast to the baseline assessment, which took place at school, the students were invited by email for the 6-month follow-up measurement."

4b-i) Report if outcomes were (self-)assessed through online questionnaires

"The respondents used their unique login information -provided by their teachers- to access the intervention at school. Students in the experimental condition received their feedback on the computer screen immediately after filling out the questionnaire."

4b-ii) Report how institutional affiliations are displayed

n/a

5) CONSORT: Describe the interventions for each group with sufficient details to allow replication, including how and when they were actually administered

5-i) Mention names, credential, affiliations of the developers, sponsors, and owners

n/a

5-ii) Describe the history/development process

"The intervention presented in this study was an improved version of the Smoke Alert intervention that was described in an earlier study [36] […]The previous version of Smoke Alert [36] was revised on the basis of focus group discussions with adolescents […]"

5-iii) Revisions and updating

"The previous version of Smoke Alert [36] was revised on the basis of focus group discussions with adolescents suggesting improvements such as the use of avatars, another web design and less extensive feedback messages. In order to increase recruitment and retention of adolescents, the revised version contained a combination of textual information with other (content-related) elements like graphics and animated videos [40-42]. […] Pilot tests revealed that the questionnaire should not be too long, resulting in a questionnaire that focused on assessing socio-demographics (age, gender, and education level), intention to start smoking and motivational determinants."

5-iv) Quality assurance methods

n/a

5-v) Ensure replicability by publishing the source code, and/or providing screenshots/screen-capture video, and/or providing flowcharts of the algorithms used

We included a screen shot of the homepage in the manuscript.

5-vi) Digital preservation

The URL of the intervention is www.smokealert.nl. The website is archived at http://www.webcitation.org/6LObMw3nP.

5-vii) Access

The teachers received an email containing a link to either the intervention website (experimental condition) or a web-based smoking questionnaire (control condition). "The teachers were informed by email about their allocation to either the experimental or control condition, with unique login information for each student attached." The login information was automatically generated by computer software that was especially developed for the execution of web-based computer-tailored programs. "The respondents used their unique login information -provided by their teachers- to access the intervention at school." Participation was free of charge. For a routine application outside of this RCT setting, there is open (and free) access to the Smoke Alert intervention.

5-viii) Mode of delivery, features/functionalities/components of the intervention and comparator, and the theoretical framework

"Smoke Alert was based on the I-Change Model, or the Integrated Model for exploring motivational and behavioral change [38, 39]." […] the revised version contained a combination of textual information with other (content-related) elements like graphics and animated videos [40-42]. […] resulting in a questionnaire that focused on assessing socio-demographics (age, gender, and education level), intention to start smoking and motivational determinants.[…] Three social cognitive concepts were measured according to the I-Change Model: namely attitude towards smoking, perceived social influence and self-efficacy not to smoke. […] Finally, non-smokers were asked to indicate to what extent they planned on using certain strategies when someone would offer a cigarette. […] Students in the experimental condition received their feedback on the computer screen immediately after filling out the questionnaire. The advice consisted of a homepage, containing an introduction and a short animated video of 30 seconds, and several subpages, each providing feedback on a specific determinant (for a screenshot of the homepage see Multimedia Appendix 1). Facts and figures were depicted on the right and left sides of the pages, also tailored to the answers of the adolescents. The introduction consisted of a personal greeting that contained the name of the student, followed by a confirmation of their smokefree status and intention to start smoking. […] The subpages of the advice addressed the psychosocial determinants. […] A copy of the advice (a PDF-file) was sent when an email address was voluntarily provided."

5-ix) Describe use parameters

n/a

5-x) Clarify the level of human involvement

For this trial, the teachers briefly introduced participation to the students before accessing the website and providing them with login information. There is no human involvement required for a routine application outside of this RCT setting.

5-xi) Report any prompts/reminders used

"After two email reminders 712 participants completed the follow-up questionnaires. Non-responding students received an invitation by email to briefly indicate their current smoking status by selecting a statement that best described their behavior."

5-xii) Describe any co-interventions (incl. training/support)

Our intervention was a stand-alone intervention.

6a) CONSORT: Completely defined pre-specified primary and secondary outcome measures, including how and when they were assessed

"The primary outcome measure was smoking behavior defined as smoking at least occasionally. Respondents were asked to pick a statement that best described them
out of 9 smoking-related statements [9, 17, 36, 45]. They were categorised as non-smokers if they selected one of the following statements: (1) "I have never smoked a puff"; (2) "I have tried smoking but I do not do this anymore"; (3) "I have stopped smoking, I used to smoke less than once a week" or (4) "I have stopped smoking, I used to smoke more than once a week". Respondents were categorised as smokers if they selected one of the following statements: (5) "I try smoking sometimes"; (6) "I smoke less than once a month"; (7) "I smoke at least once a month, but not weekly"; (8) "I smoke at least once a week, but not daily" or (9) " I smoke daily". To quantify the intervention effects on smoking initiation, we assessed the percentage of baseline non-smokers that indicated to smoke at follow-up."

6a-i) Online questionnaires: describe if they were validated for online use and apply CHERRIES items to describe how the questionnaires were designed/deployed

The questionnaires were developed in a system that was especially built for the execution of web-based computer-tailored programs.

6a-ii) Describe whether and how "use" (including intensity of use/dosage) was defined/measured/monitored

The computer software offered the possibility to view the number of respondents that visited the homepage of the questionnaire, that started filling out the questionnaire and that generated generating an advice. Moreover, we had the possibility to see the number of respondents that visited the different subpages of the advice.

6a-iii) Describe whether, how, and when qualitative feedback from participants was obtained

"The previous version of Smoke Alert [36] was revised on the basis of focus group discussions with adolescents […] Pilot tests revealed that the questionnaire should not be too long, resulting in a questionnaire that focused on […]" Besides that, both students and teachers had the possibility to email us with questions, feedback and (technical) problems.

6b) CONSORT: Any changes to trial outcomes after the trial commenced, with reasons

"After two email reminders 712 participants completed the follow-up questionnaires. Non-responding students received an invitation by email to briefly indicate their current smoking status by selecting a statement that best described their behavior."

7a) CONSORT: How sample size was determined

7a-i) Describe whether and how expected attrition was taken into account when calculating the sample size

"After adjusting for a potential 50% dropout at student level at 6 months [36], at least 1560 non-smokers had to be included in this study."

7b) CONSORT: When applicable, explanation of any interim analyses and stopping guidelines

n/a

8a) CONSORT: Method used to generate the random allocation sequence

"The schools were randomly assigned to the experimental or control condition. Randomisation was performed automatically by computer software that was especially developed for the execution of web-based computer-tailored programs [47]."

8b) CONSORT: Type of randomisation; details of any restriction (such as blocking and block size)

"cluster randomized controlled trial"

9) CONSORT: Mechanism used to implement the random allocation sequence (such as sequentially numbered containers), describing any steps taken to conceal the sequence until interventions were assigned

Randomisation is carried out by a computer-program. Mechanism is not described in the present manuscript, since it was not relevant for the present study.

10) CONSORT: Who generated the random allocation sequence, who enrolled participants, and who assigned participants to interventions

Randomisation is performed at school level and carried out by a computer-program. Mechanism is not described in the present manuscript, since it was not relevant for the present study.

11a) CONSORT: Blinding - If done, who was blinded after assignment to interventions (for example, participants, care providers, those assessing outcomes) and how

11a-i) Specify who was blinded, and who wasn't

n/a

11a-ii) Discuss e.g., whether participants knew which intervention was the "intervention of interest" and which one was the "comparator"

n/a

11b) CONSORT: If relevant, description of the similarity of interventions

n/a

12a) CONSORT: Statistical methods used to compare groups for primary and secondary outcomes

"All analyses were done using MLwiN (multilevel modelling for Windows), since adolescents were nested within schools. […] To check whether the randomisation was successful, both conditions were compared on age, gender, educational level and intention to start smoking. Dropout was checked using multilevel logistic regression analysis with attrition at posttest as outcome, and pretest demographic variables and intention to start smoking as predictors. Interaction terms of predictors with treatment condition were included in the model to analyze whether predictors for dropout differed by condition."

12a-i) Imputation techniques to deal with attrition / missing values

"To accommodate missing values in the effect analyses, the multiple imputation procedure in MLwiN was employed, the results being based on 50 imputed datasets."

12b) CONSORT: Methods for additional analyses, such as subgroup analyses and adjusted analyses

"Differences between the conditions on smoking initiation were analyzed by multilevel logistic regression analysis. Demographic variables and significant baseline differences were entered as covariates. Interactions of these covariates with treatment condition were also included to examine inequalities in the effects of the intervention on smoking initiation. Interactions with a P-value higher than .05 were deleted stepwise. Effects of covariates and the intervention were considered significant if $P \leq .05$."

RESULTS

13a) CONSORT: For each group, the numbers of participants who were randomly assigned, received intended treatment, and were analysed for the primary outcome

We added a CONSORT flowchart (Figure 2) in which these results are shown.

13b) CONSORT: For each group, losses and exclusions after randomisation, together with reasons

We added a CONSORT flowchart in which these results are shown.

13b-i) Attrition diagram

We added a CONSORT flowchart in which these results are shown.

14a) CONSORT: Dates defining the periods of recruitment and follow-up

"During the spring of 2011, 1380 secondary schools throughout the whole country were approached by sending a letter to their principals. [...] Teachers were requested to schedule half an hour, between 9 May and 10 June 2011, for the students to complete the web-based questionnaire in the classroom. In contrast to the baseline assessment, which took place at school, the students were invited by email for the 6-month follow-up measurement."

14a-i) Indicate if critical "secular events" fell into the study period

14b) CONSORT: Why the trial ended or was stopped (early)
n/a
15) CONSORT: A table showing baseline demographic and clinical characteristics for each group
See Table 2: Baseline sample characteristics of non-smoking adolescents (n=4979) recruited during the spring of 2011.
15-i) Report demographics associated with digital divide issues
See Table 2.
16a) CONSORT: For each group, number of participants (denominator) included in each analysis and whether the analysis was by original assigned groups
16-i) Report multiple "denominators" and provide definitions
See our flowchart (Figure 2).
16-ii) Primary analysis should be intent-to-treat
"To accommodate missing values in the effect analyses, the multiple imputation procedure in MLwiN was employed, the results being based on 50 imputed datasets. This procedure saves cases for the analysis, and can be considered an intention-to-treat analysis."
17a) CONSORT: For each primary and secondary outcome, results for each group, and the estimated effect size and its precision (such as 95% confidence interval)
See Table 3.
17a-i) Presentation of process outcomes such as metrics of use and intensity of use
n/a
17b) CONSORT: For binary outcomes, presentation of both absolute and relative effect sizes is recommended
See Table 3 and the results that are reported.
18) CONSORT: Results of any other analyses performed, including subgroup analyses and adjusted analyses, distinguishing pre-specified from exploratory
"Next, in order to test our second hypothesis, an analysis for the age cohort of 14-16 years was done."
18-i) Subgroup analysis of comparing only users
n/a
19) CONSORT: All important harms or unintended effects in each group
No intended harms were reported.
19-i) Include privacy breaches, technical problems
n/a
19-ii) Include qualitative feedback from participants or observations from staff/researchers
n/a

DISCUSSION

20) CONSORT: Trial limitations, addressing sources of potential bias, imprecision, multiplicity of analyses
20-i) Typical limitations in ehealth trials
"First, all measurements were self reports. Biochemical validation may not be necessary or advisable in studies like the current study using internet data collection without face-to-face contact [52]. [...]Second, of the 1380 schools approached, only 89 agreed to participate revealing an overall negative climate towards smoking prevention in the Netherlands. […] Third, we experienced high but equal loss to follow-up in both the experimental and control condition. The attrition at student level was 82% and outnumbered the expected dropout rate of 50%."
21) CONSORT: Generalisability (external validity, applicability) of the trial findings
21-i) Generalizability to other populations
"Second, of the 1380 schools approached, only 89 agreed to participate revealing an overall negative climate towards smoking prevention in the Netherlands. Most often, reasons for non-participation mentioned were lack of time and lack of interest, which is often the case in many schools in the Netherlands, since health promotion is not integrated into the Dutch school curriculum [10, 58, 59]. This clearly implies a need for health promoting policies to outline the need for the adoption of evidence based smoking prevention."
21-ii) Discuss if there were elements in the RCT that would be different in a routine application setting
In a routine application setting, the students do not need login information to access the intervention.
22) CONSORT: Interpretation consistent with results, balancing benefits and harms, and considering other relevant evidence
22-i) Restate study questions and summarize the answers suggested by the data, starting with primary outcomes and process outcomes (use)
"This paper describes a cluster randomized controlled trial examining the effectiveness of a computer tailored intervention on smoking prevention, called Smoke Alert, aimed at adolescents. This trial was conducted among students aged 10-20 years in order to detect whether implementation could be recommended for a wide age range of students. We hypothesized that smoking initiation rates would be lower in the experimental condition at 6-month follow-up, as compared to the control condition. The results offered some support for our first hypothesis revealing that students in the control condition reported higher smoking initiation at 6-month follow-up. The results provided significant support for our second hypothesis, as the data for the 14-16 age group showed a significant effect with lower smoking initiation rates in the experimental condition."
22-ii) Highlight unanswered new questions, suggest future research
"Further research is needed to focus on effects of the Smoke Alert program in the years following program delivery. [...] It is important to gain insight into predictors of dropout and examine strategies to enhance engagement with web-based interventions over time and reduce the excessive rates of attrition. Web 2.0 features, like allowing adolescents to manage, display, and share their data with peers, could be incorporated in order to attract, retain, and engage adolescents [62, 64], although these hypotheses require additional research."

Other information

23) CONSORT: Registration number and name of trial registry
International Standard Randomized Controlled Trial Number (ISRCTN): 77864351.
24) CONSORT: Where the full trial protocol can be accessed, if available
n/a
25) CONSORT: Sources of funding and other support (such as supply of drugs), role of funders
"This study was supported by grants from the Dutch Ministry of Health, Welfare and Sport."
X26-i) Comment on ethics committee approval
"Students participation in both conditions was voluntary, respondents were guaranteed anonymity and were explained that they could withdraw participation at any moment. This study was part of a larger study on the effectiveness of the Smoke Alert study for which ethical clearance was obtained [36]."
x26-ii) Outline informed consent procedures
" Furthermore, a letter to inform the students' parents was attached."

X26-iii) Safety and security procedures
n/a
X27-i) State the relation of the study team towards the system being evaluated
"Hein de Vries is scientific director of Vision2Health, a company that licenses evidence-based innovative computer-tailored health communication tools."
